# Cophylogenetic relationships between *Anicetus* parasitoids (Hymenoptera: Encyrtidae) and their scale insect hosts (Hemiptera: Coccidae)

**DOI:** 10.1186/1471-2148-13-275

**Published:** 2013-12-23

**Authors:** Jun Deng, Fang Yu, Hai-Bin Li, Marco Gebiola, Yves Desdevises, San-An Wu, Yan-Zhou Zhang

**Affiliations:** 1 Key Laboratory of Zoological Systematics and Evolution, Institute of Zoology, Chinese Academy of Sciences, Beijing 100101, China; 2 Key Laboratory for Silviculture and Conservation of Ministry of Education, Beijing Forestry University, Beijing 100083, China; 3 CNR,– Istituto per la Protezione delle Piante, UOS di Portici, Via Università 133, 80055 Portici (NA), Italy; 4 Department of Entomology, The University of Arizona, 410 Forbes Building, Tucson, AZ 85721, USA; 5 Sorbonne Universités, UPMC Univ Paris 06, UMR 7232, Integrative Biology of Marine Organisms, Observatoire Océanologique, F-66650 Banyuls/Mer, France; 6 CNRS, UMR 7232, Integrative Biology of Marine Organisms, Observatoire Océanologique, F-66650 Banyuls/Mer, France

**Keywords:** Host-parasitoid interactions, Sorting, Speciation, COI, 28S-D2

## Abstract

**Background:**

Numerous studies have investigated cospeciation between parasites and their hosts, but there have been few studies concerning parasitoids and insect hosts. The high diversity and host specialization observed in *Anicetus* species suggest that speciation and adaptive radiation might take place with species diversification in scale insect hosts. Here we examined the evolutionary history of the association between *Anicetus* species and their scale insect hosts via distance-based and tree-based methods.

**Results:**

A total of 94 *Anicetus* individuals (nine parasitoid species) and 113 scale insect individuals (seven host species) from 14 provinces in China were collected in the present study. DNA sequence data from a mitochondrial gene (COI) and a nuclear ribosomal gene (28S D2 region) were used to reconstruct the phylogenies of *Anicetus* species and their hosts. The distance-based analysis showed a significant fit between *Anicetus* species and their hosts, but tree-based analyses suggested that this significant signal could be observed only when the cost of host-switching was high, indicating the presence of parasite sorting on related host species.

**Conclusions:**

This study, based on extensive rearing of parasitoids and species identification, provides strong evidence for a prevalence of sorting events and high host specificity in the genus *Anicetus*, offering insights into the diversification process of *Anicetus* species parasitizing scale insects.

## Background

The study of the evolution of host-parasite associations has a long history, with the first paper published a century ago [1-6]. Since then, numerous host-symbiont systems have been observed and several analytical methods proposed. When the host and parasite phylogenetic trees are the same, that is when visual inspection show that the two trees precisely match, with hosts and corresponding parasites at the same positions, a cospeciation pattern can be directly inferred. In other situations, the reconstruction of a hypothetical coevolutionary scenario is not straightforward, as it can involve different events including cospeciation, duplication, lineage sorting and host-switching [7]. In such cases, a rigorous and specific method must be used to differentiate cospeciation from a number of potential scenarios.

In the last two decades, several methods were developed to assess the level of cospeciation in symbiotic associations [8], and the availability of programs such as TreeMap [9], TreeFitter [10,11] and ParaFit [12] has led to an increased level of accuracy in host-parasite cospeciation studies [13-15]. These software search for an optimal evolutionary scenario for the association between hosts and their symbionts (for example, parasites). Previous work has investigated cospeciation between parasites and their hosts, such as lice and mammals [16-21], plants and insects [22-25], plants and fungi [26], fish and Platyhelminthes [7,27,28], and animals and viruses [29,30]. However, cophylogeny between parasitoids and their insect hosts has been rarely investigated, with the few previous studies focusing on Lepidoptera-parasitoids systems [31,32].

Almost every plant-feeding insect species is attacked by at least one parasitoid species [33] and even without strict host specificity, there are at least as many (and possibly more) parasites than free-living species. Among Hymenopteran parasitoids, Encyrtidae (Hymenoptera: Chalcidoidea) is an economically important group of nearly 4000 species of natural enemies of Lepidoptera, scale insects and other insect orders [34]. The genus *Anicetus* Howard is well known for its important economic significance. Several *Anicetus* species, such as *A. beneficus* Ishii & Yasumatsu, are frequently used as biological control agents of wax and soft scales of the genus *Ceroplastes* Gray (Homoptera: Coccidae), which are significant pests of important agricultural crops [35-37]. However, due to their small size and frequent lack of distinct morphological characters, the accurate identification of wax scales and parasitoids is still a great challenge for taxonomists. The study of cophylogenetic patterns between species of *Anicetus* and *Ceroplastes* is therefore difficult, however, it is also crucial for a better understanding of speciation and diversification processes in this parasitoid genus. Two recent DNA barcoding studies of *Anicetus* and their wax scale hosts were used as a taxonomic reference for the present study [38,39].

Several recent DNA-based studies strongly suggest that morphologically similar lineages traditionally considered as single species are instead genetically isolated, and in many cases host-specific [40-43]. Koinobiont parasitic Hymenoptera, in particular, display an intricate physiological relationship with their hosts and consequently tend to have relatively narrow host ranges [44]. The degree of host specificity of Encyrtidae is variable. For example, *Anagyrus sp. nov*. nr. *sinope* and *Leptomastix dactylopii* Howard are two parasitoids of mealybug species; the former is highly host specific, whereas the latter displays a wider host range, having been recorded from more than 20 host species [45]. Some Encyrtidae species such as *Copidosoma floridanum* (Ashmead) [46] exclusively parasitize a given host family or subfamily, while other *Copidosoma* species have a wider host range and attack different families of Lepidoptera [47]. High host specificity has been reported in *Comperia merceti* (Compere) [48], *Gyranusoidea tebygi* Noyes [49,50], and more recently in *Encyrtus sasakii*[51]. Zhang et al. [38] recently showed that host specificity tends to be strict in the *Anicetus* group, where species are usually restricted to one host species. Furthermore, *Anicetus* species have a low mobility and individuals that leave the host die within a few hours or days, hence they are totally reliant upon their hosts for survival. This makes the genus *Anicetus* a good candidate for evolution via cospeciation with their insect hosts.

The nine *Anicetus* species used for this study exhibit narrow host ranges and only parasitize wax scales. A large number of *Ceroplastes* individuals were collected throughout China (see Materials and methods). The aims of this study were to reconstruct molecular phylogenies for wax scale insects and their *Anicetus* parasitoids, and to assess the degree of cospeciation in this host-parasitoid association in order to better understand the drivers of species diversification in this group of parasitoids.

## Results

### Phylogenetic analyses

**Figure 1:**
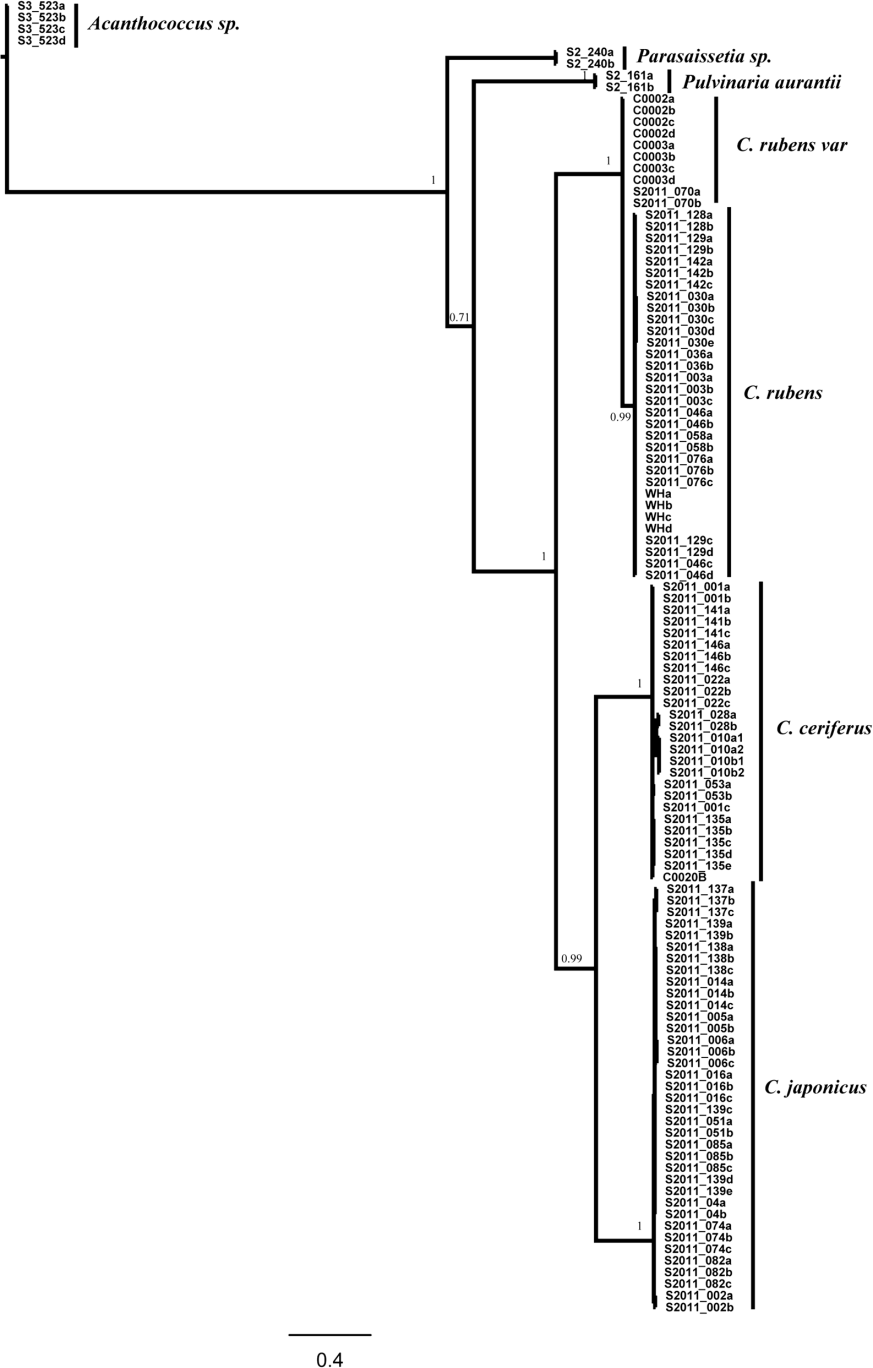
**Bayesian trees of scale insect species based on combined COI and 28S data.** Support values (posterior probabilities) are provided for each node.

**Figure 2:**
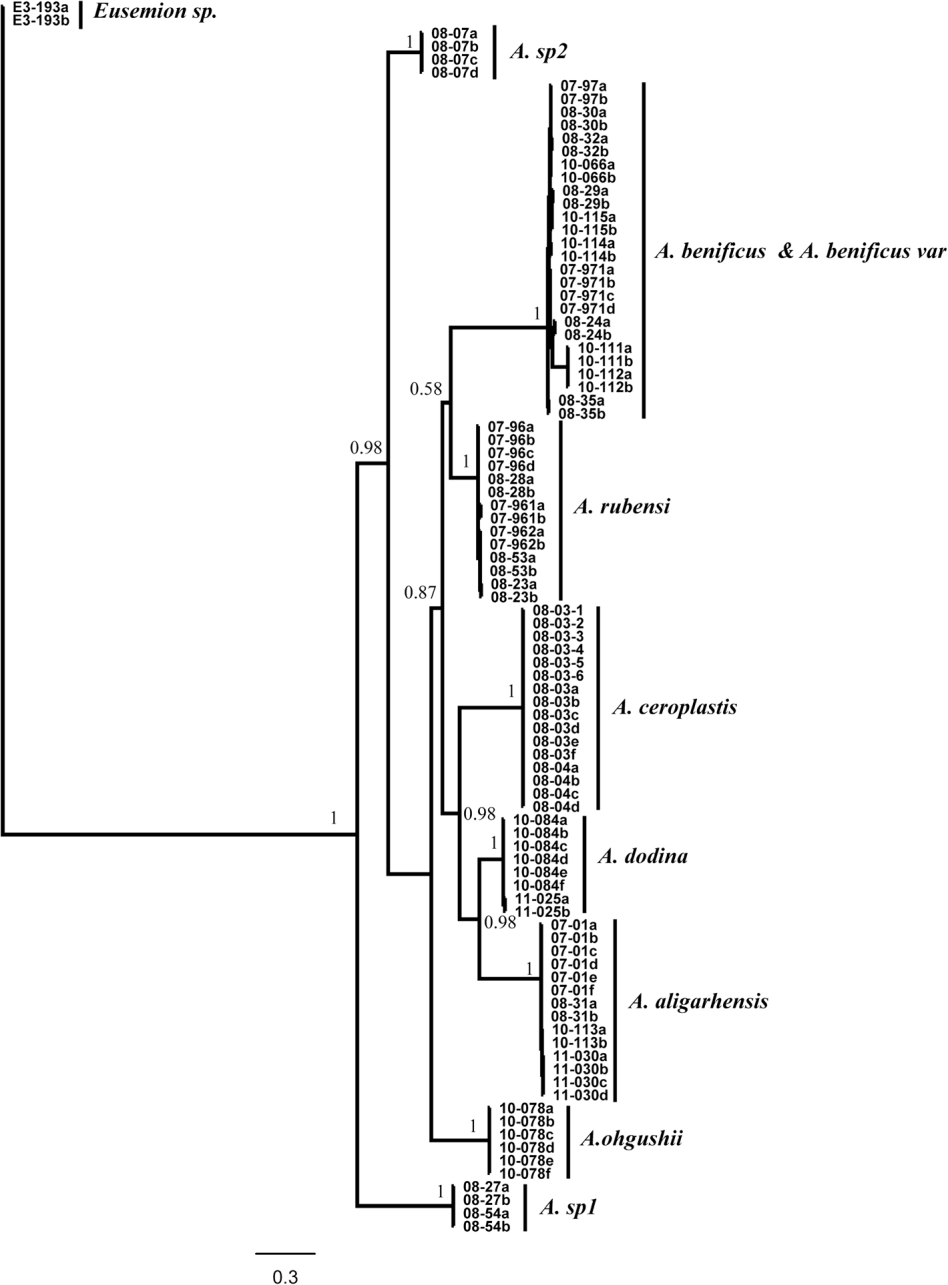
**Bayesian trees of *Anicetus *species based on combined COI and 28S data.** Support values (posterior probabilities) are provided for each node.

The partition homogeneity test indicated that the COI and 28S datasets did not display any significant signal of heterogeneity (P = 0.35 for host dataset and P = 0.66 for parasitoids dataset). This test compared the summed lengths of most-parsimonious trees computed from each dataset (i.e. gene) to the lengths of trees generated from random partitions of the combined sequences of both genes [52], and calculated the probability of obtaining a random tree similar or shorter to the length of observed summed tree. The two datasets were then combined for subsequent phylogenetic analysis. In the host tree, *Parasaissetia* sp. was strongly supported as basal clade and *Pulvinaria aurantii* was sister group to the clade of all *Ceroplastes* species, which was strongly supported (Figure 1). For parasitoids, most *Anicetus* species were strongly supported except for two groups of *A. benificus* and *A. rubensi* individuals (PP = 0.58) (Figure 2). These two species are morphologically very similar, reflecting the taxonomic uncertainty at this level.

**Figure 3:**
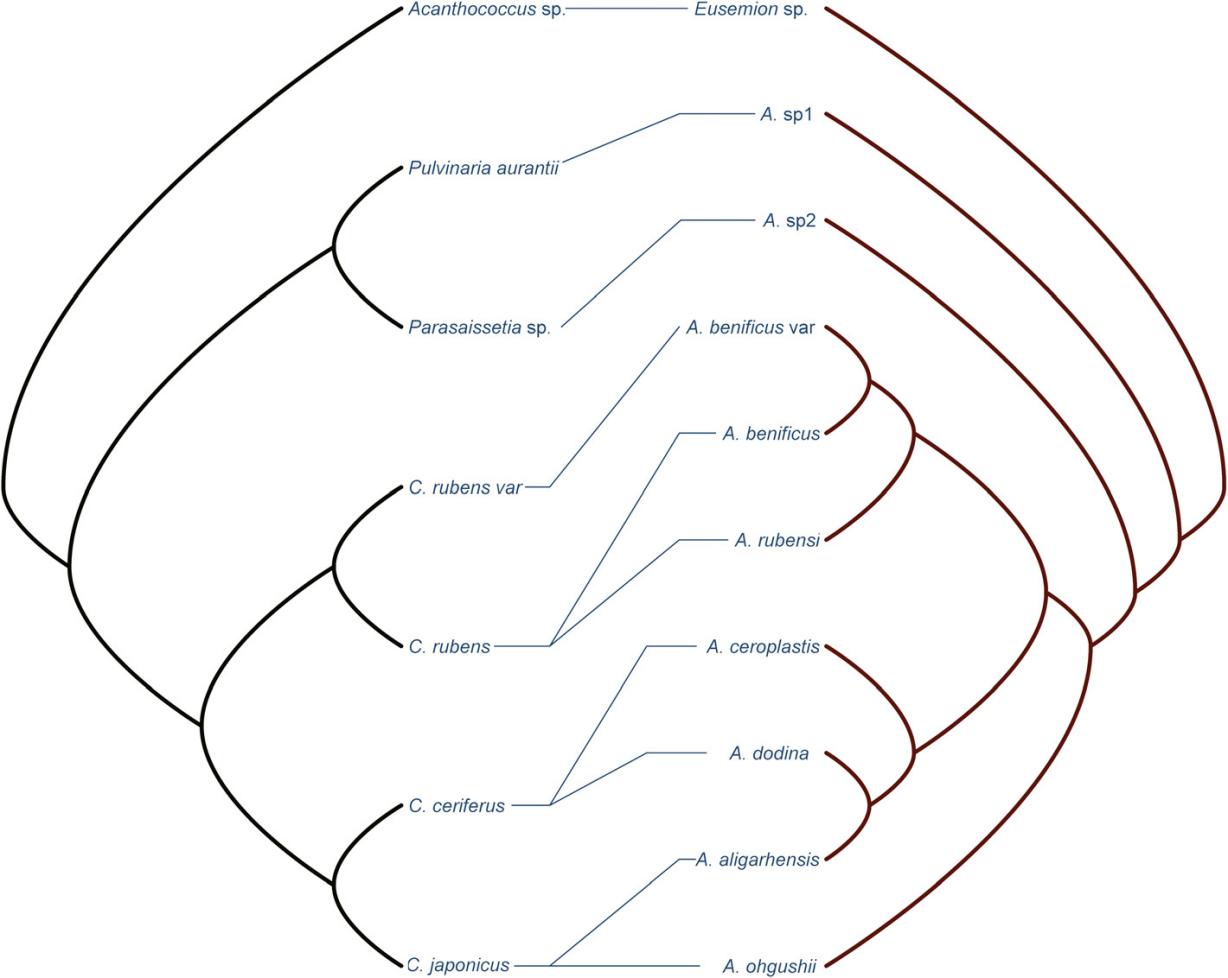
**Tanglegram between parasite (right) and host phylogenies (left) reconstructed from COI and 28S data.** Lines connecting scale insects and parasitoids indicate the pattern of host specificity.

The parasite and host phylogenies built from consensus sequences were used to assess their phylogenetic congruence (Figure 3). These trees, using consensus sequences, were identical to previous phylogenies (Figure 1, Figure 2). Furthermore, not all parasitoids from the same host clustered in the same clade, for example, *A. dodonia* Ferrière and *A. aligarhensis* Hayat, Alam & Agarval clustered together even though they use different hosts.

### Topology-based analyses: Treemap 3.0β and Jane 4

**Figure 4:**
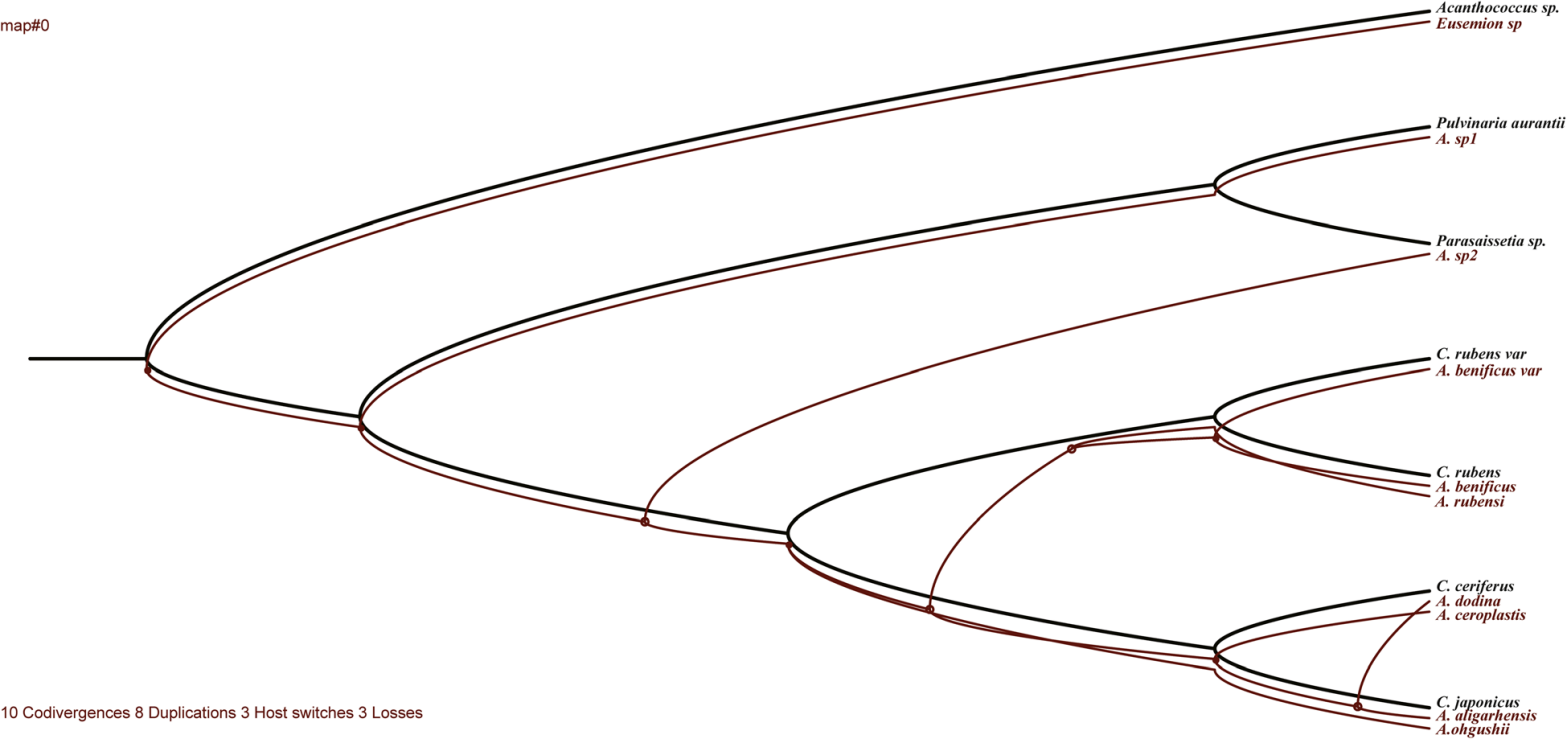
**One of the 64 optimal cophylogenetic scenarios between the *Anicetus *tree and their hosts’ tree from TreeMap 3.0β (10 codivergences, 8 switches, 3 duplications, 3 losses, total cost = 7).** Black and red lines represent hosts and their parasitoids, respectively.

**Figure 5:**
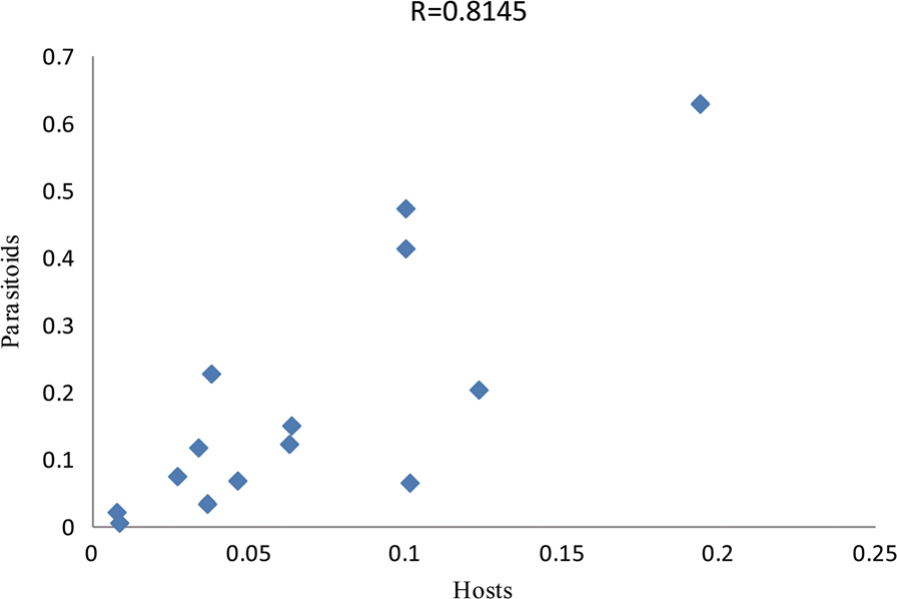
**Relationship between patristic distances of copaths for *Anicetus *** **species and their scale insect hosts.**

The tanglegram built from the phylogenetic trees and individual associations between *Anicetus* species and their scale insect hosts (Figure 3) suggested that the trees did not perfectly match. We then used Treemap 3.0β that generated 64 optimal solutions to reconcile the two trees with the lowest number of coevolutionary events considering their costs (Figure 4), none of which indicated significant congruence. We used different cost sets for each of these coevolutionary events to produce different results in Jane 4 (Table 1). In both methods, each event is given a cost inversely related to the likelihood of that event [53], and a global cost is computed by summing the costs of all events needed to fit the parasitoid tree onto the host tree (i.e. tree reconciliation). A significant global cost (P = 0.004) was only observed in Jane with the TreeFitter default cost model, that is 5 for cospeciation, 4 for duplication, 0 for host-switch, 7 for loss and 0 for failure to diverge. Setting the costs of host-switch to high values in the TreeFitter default model caused the overall fit to become significant, suggesting that host-switch is rare in this host-parasitoid system. Meanwhile, a large number of sorting events (7) were found with the TreeFitter default model, in contrast to 0–1 sorting events with the other models. In addition, we compared the patristic distances (phylogenetic divergence) between parasitoid and hosts in copaths using TreeMap (Figure 5), to assess whether branch lengths are correlated in cospeciating hosts and parasitoids (corresponding branches in the two trees are called "copaths"). A strong positive correlation would support cospeciation, and in this case the slope of the linear relationship indicated the relative evolutionary rates in hosts and parasitoids because the same genes were used to build the phylogenies. The branch length randomization test suggested a strong significant correlation between copaths (r = 0.8145), supporting the hypothesis that cospeciation has occurred in this host-parasitoid association. The slope of the linear relationship using the reduced major axis method was 3.6, suggesting that *Anicetus* species have evolved more rapidly than their scale insect hosts. This result is consistent with previous results obtained for *Achrysocharoides* (Hymenoptera: Eulophidae) [32].

**Table 1:** **Results of cophylogenetic analyses with Jane for *Anicetus *** **and their hosts**

**Model**	**Event costs**	**Total cost**	**Cospeciation**	**Duplication**	**Host switch**	**Sorting event**	**Failure to diverge**	**P-value**
**Jane default model**	**01211**	**10**	**4**	**1**	**4**	**1**	**0**	**0.22**
**TreeMap default model**	**01111**	**6**	**3**	**1**	**5**	**0**	**0**	**0.53**
**TreeFitter default model**	**00211**	**7**	**5**	**4**	**0**	**7**	**0**	0.004*
**Host switch-adjusted TreeFitter model**	**00111**	**5**	**2**	**2**	**5**	**0**	**0**	**0.13**
**Codivergence adjusted TreeFitter model**	**10111**	**7**	**0**	**2**	**7**	**0**	**0**	**0.56**
**Equalweights**	**11111**	**9**	**0**	**0**	**9**	**0**	**0**	**1**

Asterisks indicate significance at the 1% level. Columns indicate the number of each event type necessary to reconcile host and parasite trees under different event cost schemes. Event costs are for cospeciation, duplication, host switching, sorting event, and failure to diverge, respectively. P-values were computed from 999 random reconstructions.

### Distance-based analysis: ParaFit

We used ParaFit to compare patristic distance between hosts and their corresponding parasitoids, to test the global fit between the two trees. In addition the method assesses if each individual host-parasitoid association (link) significantly contributes to the global fit, to evaluate which ones have a structuring effect. The global test indicated a significant congruence between *Anicetus* and scale insect trees (P = 0.01602). However, the test of individual links showed that not all host-parasite associations significantly contributed to this global fit: 4 out of 10 individual links were significant (*Eusemion sp*.-*Acanthococcus sp*., *A. ohgushii-C. japonicus*, *A dodina-C. ceriferus* and *A. aligarhensis*-*C. japonicus*), suggesting their structuring role in the global congruence.

## Discussion

A cophylogenetic signal is weak or absent in most host-parasite associations that have been studied to date [54-56]. However, significant cospeciation has been inferred in systems where host-switching is prevented by the asocial lifestyle of the host and the low mobility of the parasite. Examples include rodent-lice associations [6,18] and insect-symbiont systems where bacteria, needed for reproduction, are transmitted maternally [57,58]. The present study can be added to these few examples of extensive cospeciation, supported using various methods.

This study is the first to thoroughly investigate the cophylogenetic interactions between *Anicetus* and their scale insect hosts, and suggests the ubiquity of sorting events coupled with strong host specificity in the genus *Anicetus*. Nine genetically distinct species were clearly delineated in the phylogenic tree based on combined molecular data (28S-D2 and COI). *Anicetus benificus, A. benificus_var* and *A. rubensi*, all parasitoids of *C. rubens*, were found grouped in the phylogeny, which is congruent with the current taxonomy (Figure 2). Furthermore, morphological data confirmed this pattern, for example, the antennal clava and ovipositor of these three species are similar to each other [59]. However, not all *Anicetus* species from the same host were found to cluster in the phylogenetic tree: *A. aligarhensis* and *A. dodonia*, from two different hosts, appeared to cluster together as sister species with a high posterior probability value. The presence of host-switching (one daughter parasitoid lineage shifting to a distant host) or sorting events (when the parasitoid is absent, for example, has become extinct, in one of the daughter host lineages) may explain this result.

The distance-based analysis showed a strong cophylogenetic signal between *Anicetus* species and their scale insect hosts. However, tree-based analyses suggested that this signal is significant only when the cost of host-switching is high. In addition, a sharp increase in the number of sorting events was found using the TreeFitter cost model, suggesting that sorting has been an important component of *Anicetus* diversification. Paterson et al. [14] have suggested that three processes can lead to the absence of parasites from their hosts: sampling error, parasite extinction and the patchy distribution of parasites (resulting in the so-called “missing the boat” process). We believe that our sampling was dense enough to strongly reduce, if not eliminate, sampling error. Our observations suggest that parasitism rates even within one species are not stable and low rates are often found in some locations. Chantos et al. [60] observed that the encyrtid wasp *Neodusmetia sangwani* (Subba Rao) exhibits a patchy geographic distribution. Our investigations showed that most *Ceroplastes* species only carried up to three *Anicetus* individuals and that a patchy distribution of *Anicetus* species may be very common in wax scales. Therefore, *Anicetus* species may have been absent from the host founder population because of a patchy distribution and the small size of the host population when speciation took place, leading to a sorting event via a “missing the boat” process. In addition, host specific parasites are likely to possess fewer populations than multi-host parasites [56]. These observations support the conclusion that some parasites in this study may have gone extinct from a host lineage after a host speciation event.

In the present study, we observed that *Anicetus* species only attacked and parasitized single host species. This is coherent with the hypothesis that the evolution of obligate parasites (or parasitoids) with limited ability to transfer between different host species is tightly linked to the evolution of their own host species [61]. However, the congruence of host-parasite phylogenies is not perfect, which can be explained by a mix of coevolutionary events such as host switching, parasite speciation without host speciation (duplication), parasite extinction, and non-colonization of all host lineages [62]. A previous study suggested that *Anicetus* species is adapted to narrow niches or restricted to particular hosts. Specifically, *A. ceroplastis*, *A. beneficus*, *A. rubensi and A. aligarhensis* develop on the same host (*Ceroplastes spp.*), and thus far they have not been reared from other hosts across China [38]. After investigating a high number of samples from different provinces, we found that these species and others display strict host specificity (Table 2). For example, *A.* sp1 and *A.* sp2 were observed to only attack *Pulvinaria aurantii* and *Parasaissetia* sp., respectively. This host specificity is not congruent with former multi-host records of the genus *Anicetus* observed in previous studies [63-65], which could be explained by the extensive examination carried out in the present study, coupled with the use of molecular data.

**Table 2:** **A detailed description of host specificity of each *Anicetus *** **species**

***Anicetus *** **species**	**Location**	**Host**	**Date**
*Eusemion* sp.	Guangxi, baise	*Acanthococcus* sp.	2.vi.2013
*A.* sp2	Fujian, Nanjing	*Parasaissetia* sp.	23.ix.2008
*A.* sp1	Shanghai	*pulvinaria aurantii*	19.v.2008
*A. aligarhensis*	Shanxi, Taiyuan	*C. japonicus*	3.vi.2007
*A. aligarhensis*	Hubei, Jingzhou	*C. japonicus*	10.v.2011
*A. aligarhensis*	Hubei, Xiangyang	*C. japonicus*	15.viii.2011
*A. ohgushii*	Zhejiang, Yuyao	*C. japonicus*	29.xii.2010
*A. dodonia*	Anhui, Wuhu	*C. ceriferus*	8.vi.2010
*A. ceroplastis*	Beijing	*C. ceriferus*	15.ix.2008
*A. rubensi*	Shanghai	*C. rubens*	11.v.2008
*A. rubensi*	Jiangxi, Yichun	*C. rubens*	13.v.2009
*A. rubensi*	Jiangxi, Xinyu	*C. rubens*	15.xi.2008
*A. rubensi*	Hunan, Changsha	*C. rubens*	11.xi.2006
*A. beneficus*	Shanghai	*C. rubens*	11.v.2008
*A. beneficus*	Jiangxi, Yichun	*C. rubens*	13.xi.2008
*A. beneficus*	Hangzhou	*C. rubens*	24.ix.2009
*A. beneficus*	Sichuan, Chengdu	*C. rubens*	16.v.2009
*A. beneficus*	Austrailia	*C. rubens*	15.xi.2010
*A. beneficus*	Hangzhou	*C. rubens*	24.xi.2008
*A. beneficus*	Anhui, HeFei	*C. rubens*	20.v.2011
*A. beneficus*	Jiangxi, Xinyu	*C. rubens*	20.xi.2009
*A. beneficus*	Jiangsu, Nanjing	*C. rubens*	9.x.2009
*A. beneficus var*	Yunnan, Kunming	*C. rubens*	26.iv.2011

Many studies have supported the hypothesis that koinobionts are more host-specific than idiobionts [66-68], and a high degree of host specificity is relatively common among parasitic Hymenoptera [43,51,69]. Traditional species of parasitoid wasps that use many different hosts for their larvae can be complexes of cryptic taxa, each of them adapted to use only a few hosts [69]. An increasing number of studies using molecular data suggest that species traditionally considered generalists are in fact complexes of cryptic taxa, each of them adapted to narrow niches [38,40,42,70]. To avoid such problematic species identification leading to biased patterns of host specificity, taxonomic issues such as careful species discrimination and recognition of cryptic taxa must be carefully addressed before conducting cophylogenetic studies.

## Conclusions

In this study, we carefully assessed the identity of Anicetus species parasitizing wax scales and verified the taxonomic status of their hosts using laboratory rearing. Through the distance-based analysis (ParaFit) and the topology-based analyses (TreeMap 3.0β And Jane 4), we presented strong evidence for a prevalence of sorting events and high host specificity in the genus Anicetus, offering insights into the diversification process of Anicetus species parasitizing scale insects. Our study emphasizes that extensive rearing of parasitoids and accurate identification are important for investigating coevolutionary relationships in host-parasitoid associations.

## Methods

### Sampling

All species of *Anicetus* were reared from adults or late-stage nymphs of wax scale insects (*Ceroplastes* spp.) collected in the field from 14 provinces in China. Different *Ceroplastes* species present on the same twig or leaf were isolated and kept individually in glass vials for at least 2 months to allow parasitoids to emerge. The collected parasitoids were stored in 95% ethanol for taxonomic identification and molecular study. Parasitoids were identified by author ZYZ and *Ceroplastes* hosts by author SAW. In total, we collected seven out of twelve *Anicetus* species known from China [34] and two other species tentatively named as *Anicetus* sp1 (reared form *Pulvinaria aurantii*) and *Anicetus* sp2 (reared from *Parasaissetia* sp.). Although we have collected six out of ten *Ceroplastes* species known in China [39], *Anicetus* species were reared from three of them (see Additional file 1 and Additional file 2). Voucher specimens were deposited at the Institute of Zoology, Chinese Academy of Sciences, Beijing.

### DNA extraction, amplification and sequencing

Total DNA was extracted from individuals preserved in 95% ethanol using DNeasy Blood & Tissue Kit (Qiagen), following the manufacturer’s protocol. Protocols for PCR amplification of COI and 28S followed Zhang et al. [38] for parasitoids and Deng et al. [39] for scale insects. Products were visualized on 1% agarose and the most intense products were sequenced bidirectionally using BigDye v3.1 on an ABI3730xl DNA Analyzer (Applied Biosystems). GenBank accession numbers are given in Additional file 1 and Additional file 2.

### Phylogenetic reconstruction

Sequences of COI and 28S were aligned using Clustal W 1.8.3 [71] as implemented in BioEdit 7.0.5 [72]. Some sequences of hosts and parasitoids were retrieved from previous studies [38,39]. Several samples collected from other cities in China (see electronic supplementary material, Additional file 1 and Additional file 2) were added to our data. A total of 94 *Anicetus* individuals (nine parasitoid species) and 113 scale insect individuals (seven host species) were used in the present study. To confirm that sequence data could be concatenated, the homogeneity of the COI and 28S data sets was assessed using a partition homogeneity test (100 replicates) [73] as implemented in the program PAUP* 4.0b10 [74]. We estimated the DNA sequence evolution model that best fit the data using jModelTest 0.1.1. [75], applying the Akaike Information Criterion (AIC). For the COI data, we used a codon model (nucmodel = codon, code = metmt in MrBayes, see below). For the 28S data, the selected models for hosts and parasitoids were HKY + G and GTR + G, respectively. Bayesian analyses (BA) of combined data sets were performed with MrBayes 3.2 [76] with these evolutionary models assigned separately to the respective partitions. A Markov chain Monte Carlo search was run with four chains of 10,000,000 generations sampled once every 100 generations. A plot of number of generations versus the log probability was used to check for stationarity, and posterior probability values (PP) were calculated after the first 25% of trees were discarded. To test the convergence of chains and assess stationarity of BA parameter values, the effective sample sizes (ESS) of all parameters were calculated using Tracer 1.5 [77]. Analyses of these parameters in Tracer 1.5 shown that most ESS values were exceeding 500, indicating strong equilibrium after discarding burn-in. *Eusemion* sp. (Hymenoptera: Encyrtidae) was chosen as an outgroup of *Anicetus* parastoids and *Acanthococcus* sp. (Hemiptera: Eriococcidae) as an outgroup of coccids.

### Cophylogenetic analyses

Seven host species and nine *Anicetus* species were used for cophylogenetic analyses. Consensus sequences of COI and 28S were created by collapsing all sequences from the same species using BioEdit 7.0.5, and used in the analysis of the congruence of parasite and host phylogenies. Several methods using TreeMap [9,78], TreeFitter [11], Jane 4 [79] and ParaFit [12], are available to study the congruence between symbiont and host phylogenies. In the present study, three methods were used: a distance-based method called ParaFit implemented in CopyCat [80] and topology (or tree)-based methods implemented in Jane 4 and TreeMap 3 (developed by Mike Charleston and available at http://sites.google.com/site/cophylogeny).

TreeMap is a popular topology-based program that reconciles two trees using four types of events (cospeciation (C), host-switching (H), duplication (D), and sorting (S)) to graphically depict the differences between the phylogenies [9,81]. In our study, TreeMap 3.0β was used to reconstruct the tanglegram and assess the congruence between parasite and host phylogenies (including outgroups). We also computed the correlation between evolutionary divergences in previously identified cospeciating pairs (“copaths”) in TreeMap to test whether parasitoids evolve faster than their hosts [9]. As the same genes were used to build host and parasite trees, the slope of the linear relationship between corresponding divergences reflect their relative evolutionary rates.

Jane 4 uses a polynomial time dynamic programming algorithm in conjunction with a genetic algorithm to compare the two tree topologies by optimally mapping the parasite tree onto the host tree using different event costs to find very good, and often optimal, solutions to reconcile the two phylogenetic trees [79,82]. We used Jane 4 with 100 generations and a population size of 200 as parameters of the genetic algorithm. Six different cost models were used to find the minimum total cost (see Table 1). All models were tested using random tip mappings with 100 randomizations. Jane 4 can handle polytomies, considered as soft polytomies, which are resolved in order to minimize the global cost. We selected the option "Prevent mid-polytomy" to ensure that no coevolutionary event was involved in the (very short) branches created to resolve polytomies.

ParaFit is not dependent on fully resolved phylogenies and uses matrices of phylogenetic distances for both hosts and parasites [12]. Three types of information are used to describe the situation in matrix form: a matrix of phylogenetic distances among parasites, a matrix of phylogenetic distances among hosts, and a matrix of the observed host-parasite associations. All of the combined consensus data of parasitoids and hosts were used to statistically assess the global fit between trees and the significance of the contribution of each individual link between taxa to this global congruence. Tests of significance were performed using 999 permutations.

### Availability of supporting data

GenBank accession numbers are provided in Additional file 1: Table S1 and Additional file 2: Table S2). The sequence alignments for tree construction have been deposited in the TreeBASE with accession URL (http://purl.org/phylo/treebase/phylows/study/TB2:S15010).

## Competing interests

The authors declare that they have no competing interests.

## Authors’ contributions

JD, FY and YZZ assembled all of the sequences. JD, FY, HBL, MG, YD and YZZ performed data analyses. JD, MG, YD, SAW and YZZ wrote the manuscript. All of the authors read and approved the final manuscript.

## Supplementary Material

Additional file 1: Table S1 List of scale insect samples used for molecular work. (Collectors’ names are abbreviated as follows: FPZ = Fang-Ping Zhang; GHH = Guo-Hua Huang; HBL = Hai-Bin Li; HLL = Hong-Liang Li; HL = Hu Li; JD = Jun Deng; JQW = Jian-Qin Wu; KJW = Kai-Ju Wei; NN = Nan Nan; QS = Qiang Shen; SAW = San-An Wu; SBH = Shao-Bin Huang; XHY = Xiu-Hao Yang; XL = Xian Li; YJZ = Ying-Jie Zhang; YW = Ying Wang; YZZ = Yan-Zhou Zhang; YQX = Yu-Qiang Xi). Click here for file

Additional file 2: Table S2 List of parasitoid samples used for molecular work. Specimens of parasitoids used in the study. (Collectors’ names are abbreviated as follows: JD = Jun Deng; JL = John LaSalle; DYH = Dun-Yuan Huang; HLL = Hong-Liang Li; JL = Jie Li; SAW = San-An Wu; YZZ = Yan-Zhou Zhang). Click here for file
